# Successful treatment of pityriasis rubra pilaris with brodalumab after nonresponse to IL-17A inhibition: A role for the innate cytokine IL-17C

**DOI:** 10.1016/j.jdcr.2024.04.010

**Published:** 2024-04-16

**Authors:** Kailyn Valido, Michael J. Murphy, Jonathan S. Leventhal, William Damsky

**Affiliations:** a Department of Dermatology, Yale School of Medicine, New Haven, Connecticut; b Department of Pathology, Yale School of Medicine, New Haven, Connecticut

**Keywords:** brodalumab, case report, cytokines, innate immunity, interleukin-17C, medical dermatology, pathogenesis, pityriasis rubra pilaris, translational research

## Introduction

Pityriasis rubra pilaris (PRP) is an inflammatory skin disorder characterized by erythematous scaly plaques and palmoplantar keratoderma. Erythroderma is common. Studies have suggested a role for the innate keratinocyte-derived cytokine IL-17C in PRP, especially in nonresponders to IL-17A inhibition,^1^ but validation of this hypothesis in a therapeutic setting is lacking. We report a case of PRP refractory to IL-17A blockade and other therapies that was effectively treated with brodalumab (IL-17RA inhibitor) following confirmation of elevated *IL17C* expression using RNA *in situ* hybridization (RISH).

## Case report

**Fig 1:**
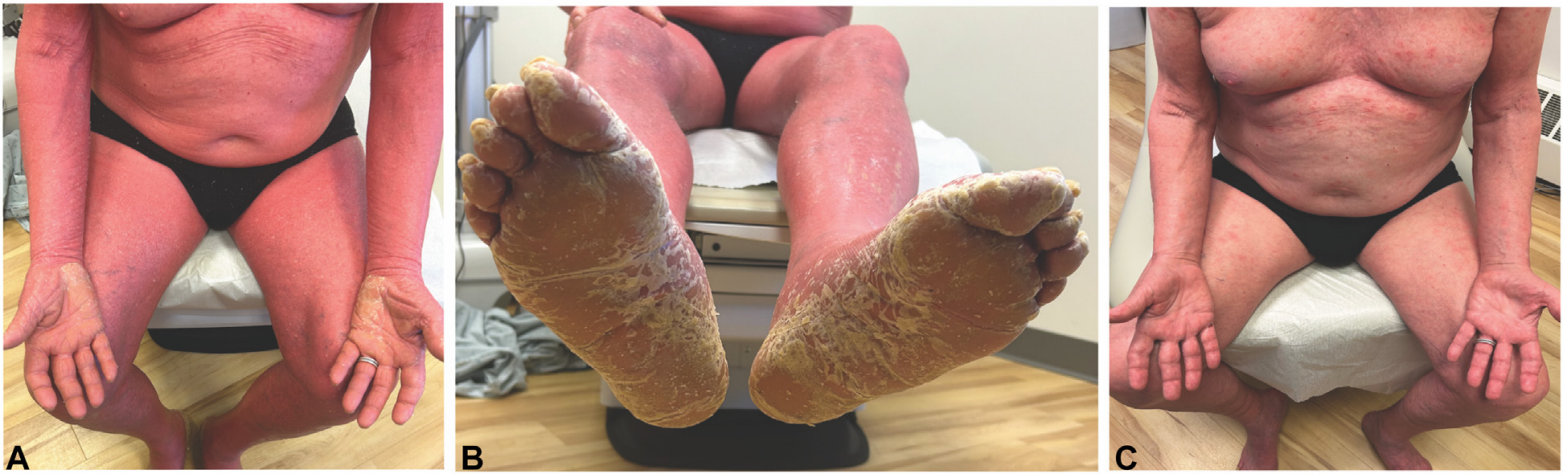
Clinical image of eruption at presentation and after 3 months of treatment with brodalumab. Upon initial presentation, erythroderma with small islands of sparing (**A**) and waxy palmoplantar keratoderma as well as nail pitting and onycholysis (**B**) and 3 months later with near complete clearance with few residual *pink* patches (**C**).

An 80-year-old man presented with a 6-month history of severe pruritus and a diffuse erythematous eruption that began on the face and spread to the trunk and extremities. On examination, the patient was erythrodermic with small islands of sparing (Fig 1, *A*). He also had bilateral palmoplantar keratoderma (Fig 1, *B*). He reported no systemic symptoms. His medical history included hypertension and benign prostatic hyperplasia, managed with chronic medications. He was previously evaluated by other dermatologists with a diagnosis of PRP versus psoriasis. Prior failed therapies included topical corticosteroids, phototherapy, guselkumab, and methotrexate (20 mg weekly). His treatment regimen upon presentation was secukinumab and upadacitinib (30 mg daily) with no improvement.

**Fig 2:**
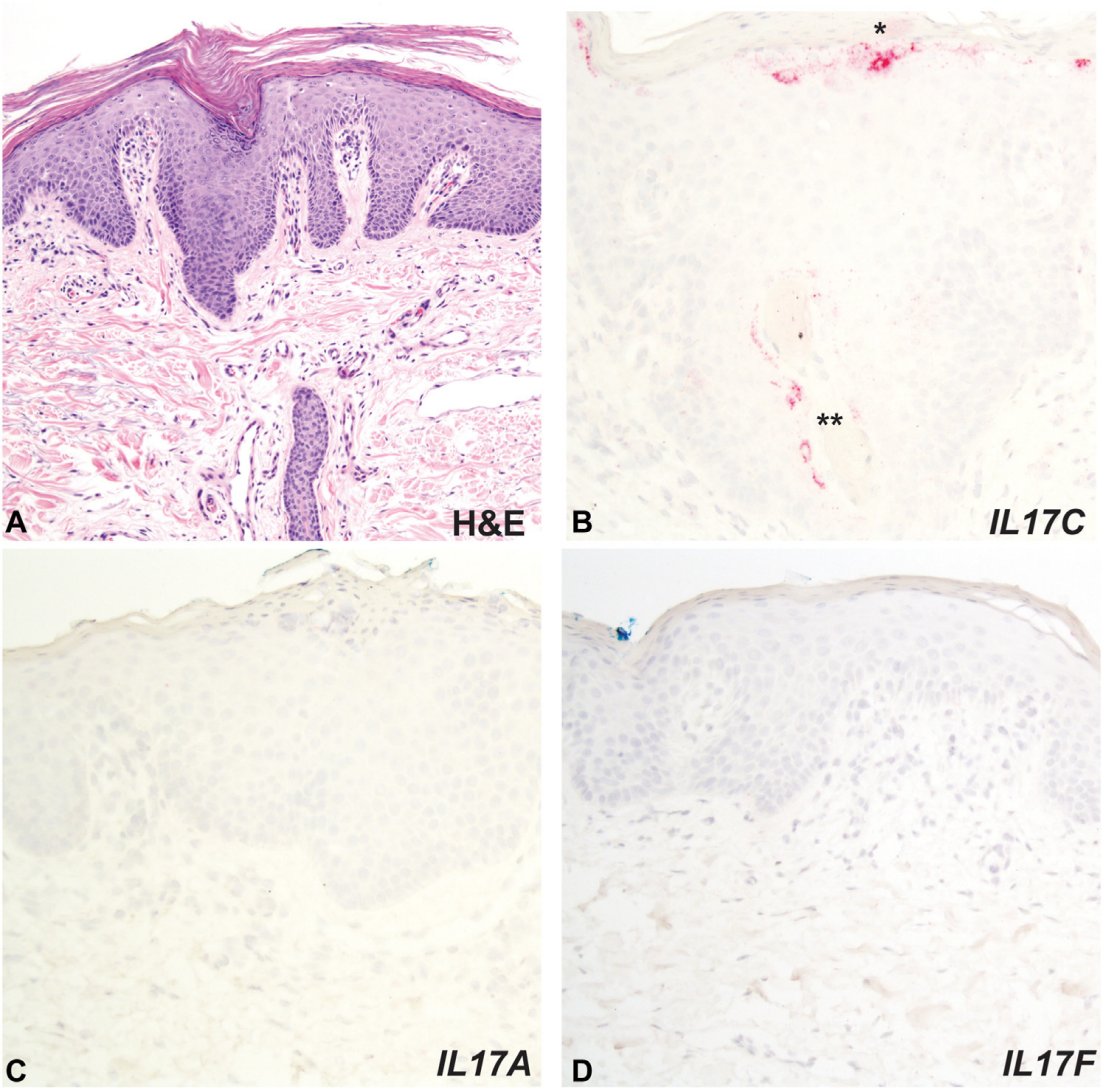
Histology and cytokine staining. **A,** A skin biopsy showed dry parakeratosis, acanthosis, and follicular plugging with a mild lymphohistiocytic inflammatory infiltrate. **B,** RISH staining (red chromogen) for *IL17C* in the upper epidermis (∗) and hair follicle (∗∗). **C,** RISH staining for *IL17A*. **D,** RISH staining for *IL17F*. *RISH*, RNA *in situ* hybridization.

A skin biopsy demonstrated acanthosis, dry parakeratosis in a checkerboard pattern, and mild inflammation typical of PRP (Fig 2, *A*). Flow cytometry of blood was unremarkable and T cell clonality was absent. To evaluate the level of *IL17C* expression compared to other therapeutically relevant cytokines given the failure of IL-17 inhibition and other therapies, we performed RISH staining on the biopsy tissue. This revealed strong expression of *IL17C*, modest expression of *IL23*, and minimal to no expression of *IL17A*, *IL17F*, and *IFNG* (Fig 2, *B*-*D*). Given the strong *IL17C* expression in this case combined with prior observations of elevated IL-17C in refractory PRP,^1^ the patient was started on brodalumab, acitretin (25 mg daily), and topical corticosteroids (triamcinolone acetonide 0.1% cream twice daily to affected areas not under occlusion). After 6 weeks, the patient noted significant skin clearing. After 3 months, there was near complete clearance of the skin and resolution of the keratoderma (Fig 1, *C*).

## Discussion

IL-17C is an innate keratinocyte-derived cytokine which signals via IL-17RA/RE. Prior studies of PRP have suggested that IL-17C is a key driver, with a roughly 167-fold upregulation at the protein level in case versus control skin.^2^ While all cases showed elevated IL-17C in that PRP cohort, nonresponders to ixekizumab tended to have relatively lower T cell derived cytokines (IL-17A/F) and elevated IL-17C. Elevated IL-17C persisted in both responders and nonresponders to ixekizumab, while IL-17A/F normalized.^2^ These observations have led some authors to speculate that the pathogenesis of PRP is grounded in innate immune activation, with the activation of Th17 immunity occurring as a secondary phenomenon. This hypothesis is further supported by the finding that <20% of patients with PRP achieve Psoriasis Area and Severity Index (PASI)-90 response to ixekizumab at 24 weeks in contrast to established PASI-90 rates of ixekizumab in psoriasis of about 72%.^3^

Brodalumab, a monoclonal antibody against IL-17RA, inhibits signaling of IL-17A/F and IL-17C complexes, whereas secukinumab and ixekizumab only inhibit IL-17A. Three prior reports of PRP treated with brodalumab all showed dramatic disease resolution.^4^, ^5^, ^6^ Our patient had a striking response to brodalumab therapy. In addition to brodalumab, he was started on concomitant acitretin and topical corticosteroids. Though topical corticosteroids could have contributed to the patient’s clinical response, the patient had previously utilized topical corticosteroids for several months with no improvement in his disease. Acitretin also could have contributed to the positive therapeutic response seen in our patient. Acitretin monotherapy, however, has demonstrated a longer time to take effect with a mean duration of 12.5 months to achieve complete clearance.^7^ Our patient had a dramatic and rapid near clinical resolution with notable skin clearing as early as 6 weeks after brodalumab initiation.

In this example of personalized medicine, we utilized cytokine staining of biopsy tissue to confirm high levels of IL-17C with a paucity of IL-17A/F correlating with nonresponse to IL-17A inhibition. Our patient also failed upadacitinib, consistent with the JAK-STAT independence of IL-17C. In addition, our patient’s nonresponse to IL-23 inhibition is also compatible with a lesser role of adaptive immunity in PRP. Here we provide proof-of-concept therapeutic evidence supporting the key role of IL-17C and innate immunity in PRP pathogenesis. Brodalumab may be an option for patients with recalcitrant disease.

## Conflicts of interest

Dr Damsky is a consultant for Pfizer, Incyte, Eli Lilly, and TWi Biotechnology, has research funding from Pfizer, Advanced Cell Diagnostics/Bio-techne, and AbbVie and receives licensing fees from EMD/Millipore/Sigma, all outside the submitted work. Authors Valido, Murphy, and Dr Leventhal have no conflicts to disclose.
